# Molecular Strain Accelerates Electron Transfer for Enhanced Oxygen Reduction

**DOI:** 10.1021/jacs.4c16637

**Published:** 2025-01-17

**Authors:** Charles B. Musgrave, Jianjun Su, Pei Xiong, Yun Song, Libei Huang, Yong Liu, Geng Li, Qiang Zhang, Yinger Xin, Molly Meng-Jung Li, Ryan Tsz Kin Kwok, Jacky W. Y. Lam, Ben Zhong Tang, William A. Goddard, Ruquan Ye

**Affiliations:** a Materials and Process Simulation Center, California Institute of Technology, Pasadena 91125, California, United States; b Department of Chemistry, State Key Laboratory of Marine Pollution, City University of Hong Kong, Hong Kong 999077, P. R. China; c Department of Applied Physics, Hong Kong Polytechnic University, Hong Kong 999077, P. R. China; d Division of Science, Engineering and Health Study, School of Professional Education and Executive Development (PolyU SPEED), The Hong Kong Polytechnic University, Hong Kong 999077, P. R. China; e Department of Chemistry and the Hong Kong Branch of Chinese National Engineering Research Center for Tissue Restoration and Reconstruction, The Hong Kong University of Science and Technology, Hong Kong 999077, China; f School of Science and Engineering, Shenzhen Institute of Aggregate Science and Technology, The Chinese University of Hong Kong, Shenzhen 518172, Guangdong, China; g City University of Hong Kong Shenzhen Research Institute, Shenzhen, Guangdong 518057, China

## Abstract

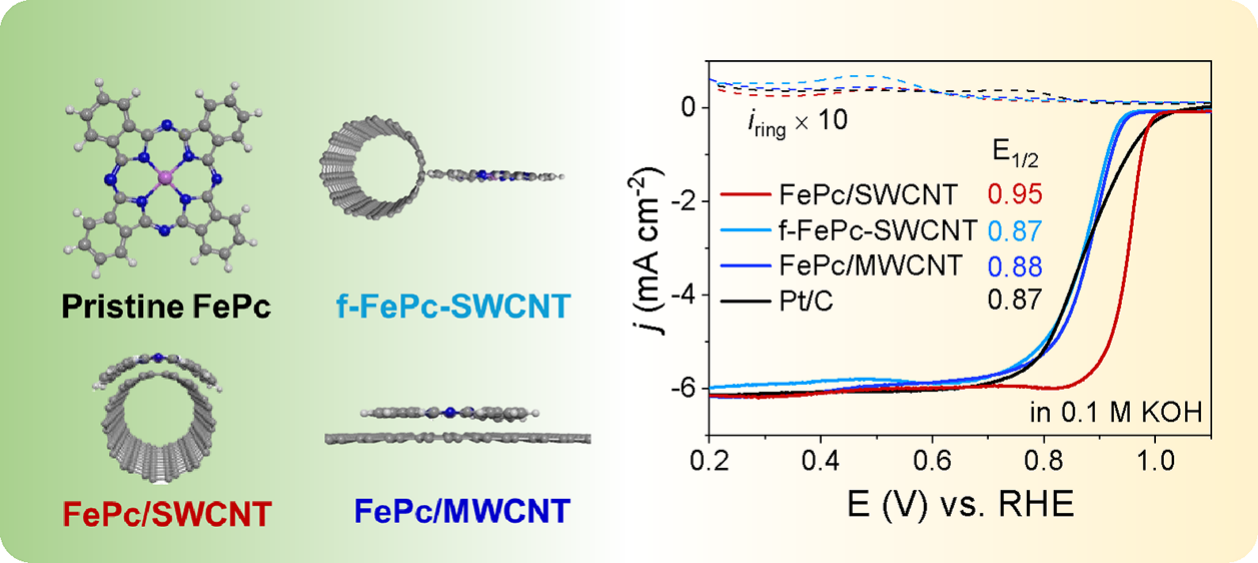

Fe–N–C materials are emerging catalysts for replacing precious platinum in the oxygen reduction reaction (ORR) for renewable energy conversion. However, their potential is hindered by sluggish ORR kinetics, leading to a high overpotential and impeding efficient energy conversion. Using iron phthalocyanine (FePc) as a model catalyst, we elucidate how the local strain can enhance the ORR performance of Fe–N–Cs. We use density functional theory to predict the reaction mechanism for the four-electron reduction of oxygen to water. Several key differences between the reaction mechanisms for curved and flat FePc suggest that molecular strain accelerates the reductive desorption of *OH by decreasing the energy barrier by ∼60 meV. Our theoretical predictions are substantiated by experimental validation; we find that strained FePc on single-walled carbon nanotubes attains a half-wave potential (*E*_1/2_) of 0.952 V versus the reversible hydrogen electrode and a Tafel slope of 35.7 mV dec^–1^, which is competitive with the best-reported Fe–N–C values. We also observe a 70 mV change in *E*_1/2_ and dramatically different Tafel slopes for the flat and curved configurations, which agree well with the calculated energies. When integrated into a zinc–air battery, our device affords a maximum power density of 350.6 mW cm^–2^ and a mass activity of 810 mAh g_Zn_^–1^ at 10 mA cm^–2^. Our results indicate that molecular strain provides a compelling tool for modulating the ORR activities of Fe–N–C materials.

## Introduction

Tremendous innovations in energy generation and utilization are necessary for achieving a sustainable future.^1−3^ In past decades, lithium-ion batteries,^4^ fuel cells,^5^ and metal–air batteries^6^ have been widely investigated, demonstrating a great potential for clean and renewable energy. Many of these technologies involve the oxygen reduction reaction (ORR), in which molecular oxygen undergoes a four-electron reduction to generate two equivalents of H_2_O, according to reaction 1.^7,8^ It is also possible for molecular oxygen to undergo a partial reaction in which a two-electron reduction generates hydrogen peroxide (H_2_O_2_, reaction 2). The equilibrium potential (*U*_0_) for the four-electron reduction is equal to 1.23 V,^9^ while *U*_0_ for the two-electron reduction is equal to 0.70 V.
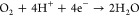
reaction 1
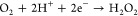
reaction 2

However, the high O=O bond energy (119 kcal mol^–1^) and slow electrochemical ORR kinetics hamper the practical application of fuel cells and metal–air batteries (i.e., Zn–air and Li–air).^10^ The industry-standard ORR catalysts require Pt group metals (PGMs), which exhibit an excellent ORR performance. However, the high cost and inferior methanol toxicity resistance preclude widespread application of these PGMs. Additionally, Pt is susceptible to durability issues, such as dissolution, sintering, and nanoparticle formation, all of which result in catalyst deactivation.^11^

Fe-based materials have shown great promise for replacing PGMs as ORR catalysts, thanks in part to their high catalytic ORR performance and relatively low cost.^12−14^ However, the preparation processes involve multistep chemical reactions and high-temperature pyrolysis. Additionally, Fe-based ORR catalysts are conventionally found in the form of Fe–N–C materials, which suffer from durability issues more so than PGMs.^15^ Regarding performance, ORR half-wave potentials (*E*_1/2_) for Fe–N–C catalysts generally fall below 0.95 V (all potentials refer to the reversible hydrogen electrode unless otherwise specified),^15,16^ such that the improvement in *E*_1/2_ is imperative.

Fe–N–Cs derived from high-temperature synthesis typically exhibit an inhomogeneous local structure, introducing complexities and uncertainties in understanding their ORR structure–activity relationship. Fe–N–C molecular analogs eliminate this structural uncertainty, making their investigation an effective alternative. Iron phthalocyanine (FePc) is a molecular catalyst that usually exhibits an *E*_1/2_ of 0.60–0.85 V^17,18^ for ORR, comparable to typical Fe–N–C catalysts. This notable similarity provides a straightforward path for delineating the ORR structure–activity relationship in Fe–N–Cs, thereby permitting the rational optimization of these materials.

Lattice strain has hitherto been applied to tailor the properties of materials, predominantly inorganic materials such as metal sulfides,^19^ metal oxides,^20^ and alloys.^21^ We recently extended the concept of strain-based modulation to molecular catalysts. We leveraged single-walled carbon nanotube (CNT) supports to form cobalt phthalocyanine (CoPc) with controlled strain, which enhanced CO adsorption and ultimately enabled efficient reduction of CO_2_ to methanol.^22^ While strained FePc has also been recently found to improve the ORR activities, the discrepancy among literature data with varying *E*_1/2_ casts doubt on the origin of improvement.^23^ In contrast to the weak binding of CO to unstrained CoPc, O_2_ binding to FePc is already strong at ambient conditions.^24^ Thus, the effect of CNT-induced strain on FePc-catalyzed ORR remains unknown.

In this work, we sought to understand the role of molecular strain on the ORR activities of FePc. Starting with theoretical calculations, we show that strained FePc improves the ORR reaction kinetics by optimizing reductive *OH desorption. By purifying the CNT precursors and optimizing the molecular loadings, we further improve the *E*_1/2_ to 0.952 V, which is competitive with the best-reported Fe–N–C values. Our comprehensive X-ray spectroscopies and Raman data show the distinct vibronic and electronic structure of strained and flat FePc induced by different molecular states, which might explain the inferior performance in literature.

## Results and Discussion

### Characterization

**Figure 1:**
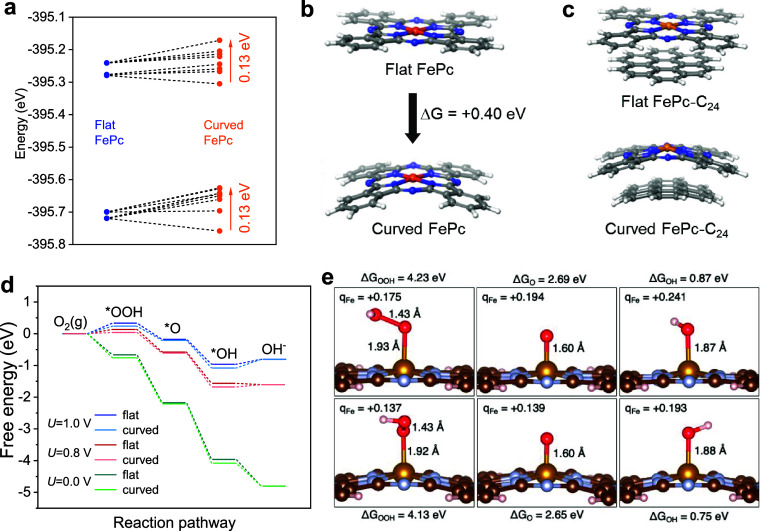
(a) Nitrogen 1s orbital energies for flat and curved FePc. (b) DFT-optimized structures for flat and curved FePc. Orange atoms are Fe, blue atoms are N, gray atoms are C, and white atoms are H. (c) Structures of flat and curved FePc-C_24_. (d) Grand canonical free energies at 298 K for flat and curved FePc-C_24_ at 0.0, +0.8, and +1.0 V. (e) DFT-optimized *OOH, *O, and *OH intermediates for flat (above) and curved (below) FePc-C_24_, accompanied by binding energies. Fe Mulliken charges are denoted by *q*_Fe_.

We begin our study by examining the impact of curvature on the FePc molecule using range-separated density functional theory (DFT; ωB97M-V with the def2-TZVP pseudopotential and basis set). This DFT methodology predicts the ground-state FePc to be a triplet (high-spin pairing of two electrons), followed by the quintet state (high-spin pairing of four electrons) at 1.02 eV, and then the singlet state (0 unpaired electron) at 1.15 eV. For the curved FePc, the triplet is still the ground state, but the quintet is 0.63 eV higher, while the singlet is 1.23 eV higher. Previous studies have reported various ground-state spins depending on the functional and basis set used.^25^ Different functionals and basis sets have also given rise to different symmetry breakings. This indicates that the ground-state FePc is susceptible to second-order Jahn–Teller distortion, which stems from nearly degenerate states interacting. In flat FePc, the four degenerate N 1s orbitals bonded to the Fe lie at −395.71 eV while the remaining four degenerate N 1s orbitals are found at −395.26 eV (Figure 1a). Upon strain distortion of the FePc, the four degenerate orbitals at −395.71 split slightly, ranging from −395.63 to −390.76 eV (∼0.13 eV deviation). The four degenerate orbitals previously found at −395.26 eV also split, ranging from −395.17 to −395.31 eV (∼0.13 eV deviation) upon molecular strain. We suspect that the N 1s orbitals belonging to N far from the distortion axis undergo little to no change. On the other hand, the N 1s orbitals close to the distortion axis undergo noticeable changes, likely because the local nuclear geometry of these N atoms is altered more dramatically (Figure 1a). We find that curving the FePc results in +0.40 eV strain (i.e., Δ*E*_flat→curved_ = +0.40 eV). Analysis of the frontier orbitals reveals that the gap between the highest occupied molecular orbital (HOMO) and the lowest unoccupied molecular orbital (LUMO) is also altered by curvature (Figure 1b). The flat FePc has a HOMO–LUMO gap of 4.60 eV, but upon curvature, the HOMO–LUMO gap increases to 4.80 eV. This is a result of the β HOMO becoming stabilized by −0.12 eV upon curvature while the β LUMO is simultaneously destabilized by +0.08 eV. The Fe Mulliken charge in the flat molecule is +0.26, but upon molecular distortion, the Fe charge drops slightly to +0.23, indicating a small amount of electron transfer from the phthalocyanine to the Fe center. We suspect that this charge decrease on Fe may translate to improved reduction kinetics.

### FePc-C_24_ Oxygen Reduction Mechanism

In a previous study on CoPc-catalyzed CO_2_ reduction, Liao et al. claimed that at −1.0 V and a slightly acidic pH of 6.8, the four outward N of the phthalocyanine are hydrogenated, forming CoPcH_4_ with a total free energy change of −2.92 eV.^26^ However, under basic conditions (pH = 13) and within the potential window for ORR (0.8–1.0 V), we do not expect FePc to undergo hydrogenations at the four outer N to form FePcH_4_. We calculated the ORR mechanism by using just the FePc molecule with the PBE-D3 DFT functional. From these preliminary calculations, we established that PBE-predicted ORR using only the FePc molecule does not yield convincing results. Although grand canonical DFT with the PBE-D3 functional has produced excellent results in other systems,^27^ the electronic structures of Fe and Fe-oxides^28^ are sometimes not captured correctly by PBE. The strongly correlated nature of the Fe d electrons is not handled properly with PBE. To address this issue, PBE+*U* is commonly used, although the addition of the on-site U parameter does not fully address the problem inherent to generalized gradient functionals like PBE.

In order to avoid these PBE problems, we investigated the ORR mechanism using the Head-Gordon ωB97M-V range-separated meta-GGA functional, which has been shown to outperform other leading functionals.^27^ We note that in the original paper Head-Gordon warns against using ωB97M-V when correlation is significant. However, recent studies utilizing this functional have demonstrated its maintained accuracy, despite Head-Gordon’s forewarning. Thus, we examined FePc-catalyzed ORR using ωB97M-V with cautious optimism. As a surrogate for the curved or strained CNT support, we use a coronene molecule (C_24_H_12_). A previous computational study on FePc ORR suggests that catalysis is moderated by an axial ligand effect.^29^ Therefore, we placed curved and flat coronenes under the curved and flat FePc molecules (FePc-C_24_) and evaluated these systems for ORR (Figure 1c).

For FePc-C_24_, DFT calculations suggest that the most facile ORR pathway is initiated by proton-coupled electron transfer (PCET) to O_2_ (g) to form the *OOH intermediate (Figure 1d,e). The *OOH intermediate then undergoes a second PCET to yield the *O intermediate and generate the first water. In the penultimate step, *O undergoes a third PCET to form the *OH intermediate. Finally, *OH undergoes a single-electron transfer (SET) to generate a free OH^–^. At 0.0 V, this mechanism is precipitously downhill, with an overall free energy change of −4.18 eV, which is expected given the substantial overpotential at 0.0 V. At 0.0 V, the curved and flat FePc-C_24_ mechanisms are essentially identical with only small deviations originating from the *OOH and *OH intermediates.

However, at 0.8 V, the flat and curved mechanisms lead to deviations. Specifically, the first PCET to convert O_2_ (g) to *OOH is nearly 0.2 eV more uphill for the flat system compared to the curved one. Additionally, the final SET that converts *OH to OH^–^ is downhill −0.04 eV for the flat catalyst yet uphill 0.07 eV for the curved catalyst.

However, at 1.0 V, the flat and curved mechanisms show a marked difference. At 1.0 V, the initial PCET to convert O_2_ (g) to *OOH is uphill 0.33 eV for the flat system and 0.24 eV for the curved FePc-C_24_ systems. Moreover, the final conversion of *OH to OH^–^ is uphill 0.15 eV for flat FePc-C_24_ and 0.27 eV for the curved analogue. At 1.0 V, the most endergonic step for the flat FePc-C_24_ is O_2_ (g) → *OOH, which is uphill 0.33 eV. At 1.0 V, the most endergonic step for the curved FePc-C_24_ is *OH → OH^–^, which is uphill 0.27 eV. The difference in the Δ*G*’s (ΔΔ*G*) for flat versus curved is 0.06 eV. The rate-limiting step for the curved FePc is the conversion of *OH to OH^–^, while the conversion of *O_2_ to *OOH is rate-limiting for the flat FePc. The observation that the most endergonic step is different for flat and curved steps implies a difference in the rate-limiting step, which suggests that there should be deviation in their Tafel slopes.

### Reaction Intermediate Adsorption Trends

**Figure 2:**
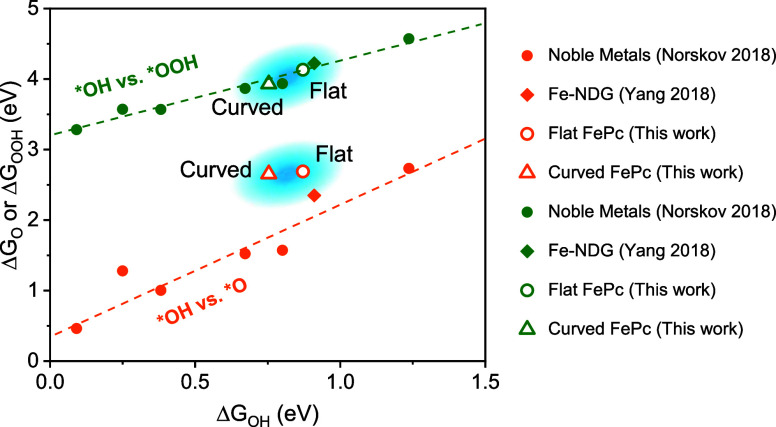
Binding energies of flat and curved FePc-C_24_ relative to conventional metal catalysts. Orange indicates *OH vs *O binding, and green indicates *OH vs *OOH binding. Closed circles represent conventional noble metals, diamonds represent Fe in nitrogen-doped graphene (Fe-NDG), open circles are flat FePc-C_24_, and open triangles are curved FePc-C_24_.

Nørskov and co-workers previously reported scaling relationships among the ORR reaction intermediates for noble metal catalysts.^30^ Scaling was originally based on the adsorption energy of the *O intermediate (Δ*G*_O_), but more modern studies have diverted attention toward the adsorption energy of *OH (Δ*G*_OH_), which typically exhibits better scaling with adsorption of *OOH (Δ*G*_OOH_). Our present calculations, along with previous calculations on Fe in nitrogen-doped graphene (Fe-NDG),^31^ show that Fe-based catalysts also follow these trends, although with some deviation from the noble metals (Figure 2).

For *OH vs *OOH scaling, the noble metals follow the relation Δ*G*_OOH_ = Δ*G*_OH_ + 3.2. We find that the Fe-based catalysts are well-behaved in this regime and abide by the same relationship. For *OH vs *O scaling, the noble metals roughly follow the relation Δ*G*_O_ = 1.7Δ*G*_OH_ + 0.4. We found no such relation for the curved and flat FePc-C_24_. The flat and curved FePc-C_24_ systems have *OH and *O binding energies that correlate positively, as was found for the noble metals, but the linear trend is different. The flat and curved FePc-C_24_ binding energies scale as Δ*G*_O_ = 0.1Δ*G*_OH_ + 2.6. This indicates that the *O binding energy is essentially independent of the *OH binding energy. We note that this linear trend is based off only 2 data points, so that we should not rely heavily on this linear regression. The well-behaved relationship between Δ*G*_OOH_ and Δ*G*_OH_ was proposed to result from the *OH and *OOH intermediates making nearly identical single bonds with the metal catalysts. Indeed, we find this to be the case for the flat and curved FePc-C_24_ systems (Figure 1e).

For the flat FePc-C_24_, *OOH makes an Fe–O bond of 1.93 Å, while *OH makes a notably shorter Fe–O bond of 1.87 Å. The *OOH and *OH binding differs slightly more for curved FePc-C_24_, which bonds to Fe–O distances of 1.92 Å for *OOH and 1.88 Å for *OH. Δ*G*_O_ is very similar for flat and curved FePc-C_24_, equaling 2.69 and 2.65 eV for the two systems, respectively. The similarity in Δ*G*_O_ is reflected by the geometry of the *O intermediate; in both the curved and flat *O intermediates, the Fe–O distance is 1.60 Å. Δ*G*_OOH_ and Δ*G*_OH_ vary more dramatically between the flat and the curved systems. Specifically, Δ*G*_OOH_ is 4.23 eV for flat FePc-C_24_ and 4.13 eV for curved. Δ*G*_OH_ values for the flat and curved FePc-C_24_ are 0.87 and 0.75 eV, respectively.

### Preparation and Characterization of Catalysts

**Figure 3:**
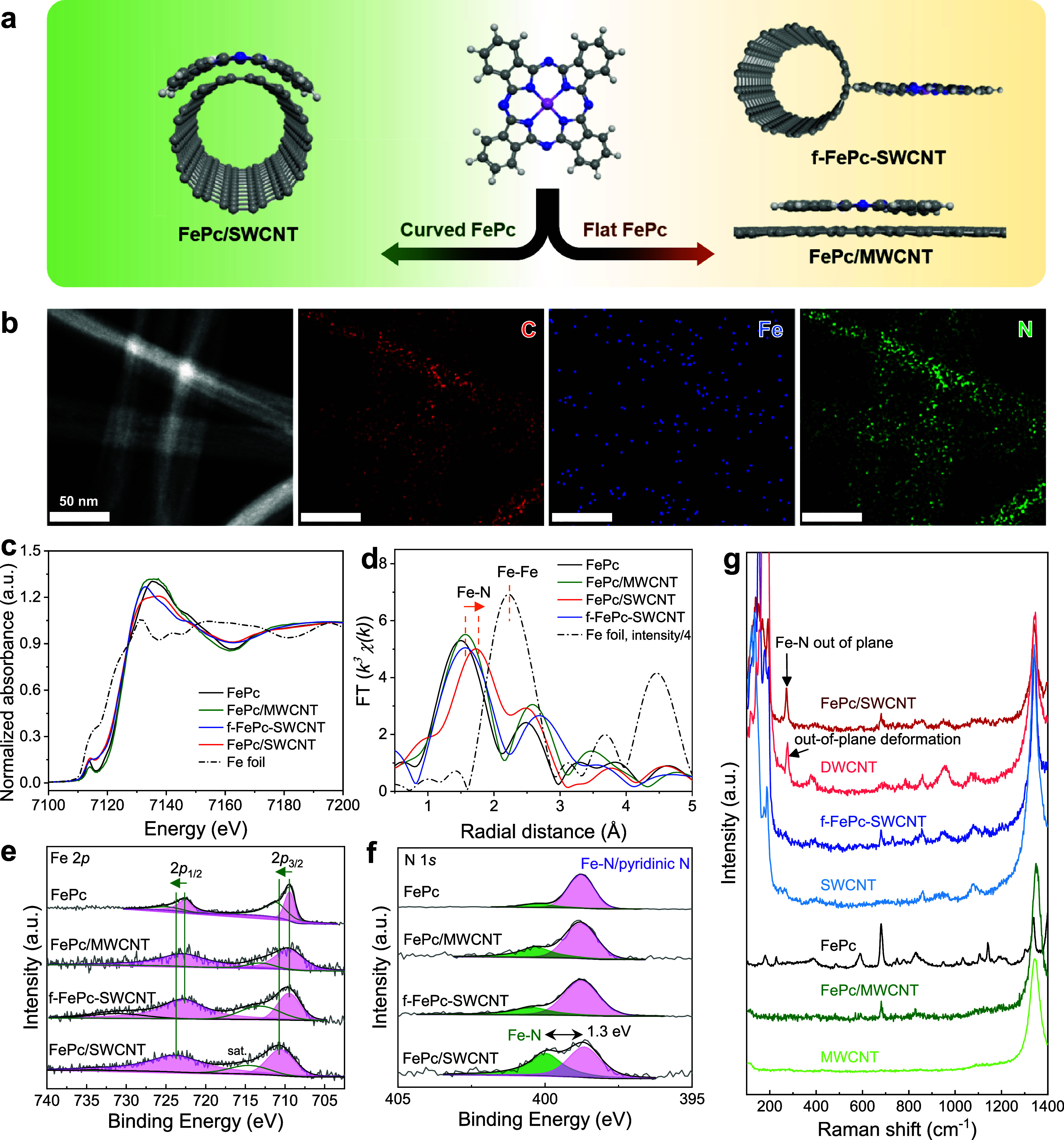
(a) Illustration of the curved and flat FePc structure; (b) elemental mapping of C, Fe, and N elements of FePc/SWCNT. (c) XANES Fe K-edge spectra, (d) Fourier-transform EXAFS, and (e) XPS of Fe 2p and (f) N 1s spectra for FePc/SWCNT, f-FePc-SWCNT, and FePc/MWCNT and FePc. (g) Raman spectra for FePc/SWCNT, f-FePc-SWCNT, FePc/MWCNT, DWCNT, SWCNT, and FePc.

To substantiate our theoretical predictions, we sought to experimentally demonstrate the advantage of strained FePc for ORR. CNTs with different diameters have been shown to be ideal substrates for inducing molecular curvature.^22,32^ We began by fabricating curved and flat FePc by depositing FePc on 1–2 nm-diameter single-walled CNTs (FePc/SWCNT) and ∼50 nm-diameter multiwalled CNTs (FePc/MWCNT), respectively. In order to eliminate the influence of the electronic structure effect of different CNTs, flat FePc on SWCNT (f-FePc-SWCNT, Figure 3a) was also synthesized via a covalent anchoring strategy.^33,34^ By improving the protocol from our former study^22^ including SWCNT purification (Figure S1) and molecular ratios (Figure S2), we further improve the ORR activities as discussed later. Figure S3 shows the SEM images of different molecular loadings of FePc/SWCNT: the high FePc loading shows obvious molecular aggregation at molecule:CNT mass ratios greater than 2:10. The formation of aggregation reduces the number of active sites, hinders the mass transfer process in the electrochemical process, and causes ORR performance degradation.^18^ We found an optimal FePc:CNT loading of 2:10, which retains the most active sites without molecular aggregation. The Fe content of all samples was set to ∼0.28 wt %, confirmed by inductively coupled plasma experiments.

The transmission electron microscopy (TEM) images show that the surface morphologies of FePc/CNT composites are similar to those of bare CNTs without obvious FePc aggregates (Figures S4 and S5). Moreover, TEM mapping images of FePc/SWCNT exhibit uniform distributions of Fe, N, and C elements on the SWCNT surface (Figure 3b). To obtain insight into the local electronic structures of Fe sites on curved and flat FePc, synchrotron radiation X-ray absorption fine structure (XAFS) analyses were performed. The pre-edge peak around 7114 eV in the Fe K-edge X-ray absorption near edge structure (XANES) is assigned to the 1s to 4p_*z*_ electronic transition of the Fe–N_4_ square planar structure (Figure 3c).^35,36^ All FePc/CNT composites show lower pre-edge intensities than the FePc standard due to a lower planar symmetry. Extended X-ray absorption fine structure (EXAFS) was performed to analyze the coordination environment of Fe sites in FePc/CNTs, leading to the simulated structures shown in Figure S6. The Fourier-transform EXAFS spectrum in R space (Figure 3d) of FePc/CNTs reveals a peak at ∼1.5 Å, corresponding to the Fe–N path.^37,38^ FePc/SWCNT presents a longer Fe–N distance than that of FePc/MWCNT and f-FePc-SWCNT, indicating a deviation in the local coordination of the Fe. The EXAFS fitting results in R space (Figures S7 and S8 and Table S1) show elongated Fe–N_1_ and Fe–C_1_ distances in FePc/SWCNT, further implying molecular distortion of the FePc moiety. Specifically, the fitting in R space shows that the Fe–N_1_ distance is ∼1.97 Å for FePc, FePc/MWCNT, and f-FePc-SWCNT, but increases to 1.998 Å for FePc/SWCNT. This result also aligns with our simulation, showing elongated Fe–N_1_ bonds from 1.955 to 1.984 Å (Table S2) and a molecular curvature of ∼12.8° for Fe/SWCNT. Conversely, FePc/MWCNT and f-FePc-SWCNT show similar coordination environments to the FePc standard. Considering that the lateral size of FePc is approximately 1.3 nm and the SWCNT has a diameter of 1–2 nm, our result suggests that the strain effect will be more significant for supports with higher local curvature.

X-ray photoelectron spectroscopy (XPS) shows that the Fe 2p and N 1s for FePc/MWCNT and f-FePc-SWCNT are similar to those of the pristine FePc molecule, indicating similar electronic structures (Figure 3e,f). All of the FePc composites show a broader Fe 2p peak compared to pristine FePc. A higher-energy Fe 2p peak shift (1.31 eV) is observed for FePc/SWCNT compared to the flat FePc in FePc/MWCNT and f-FePc-SWCNT. Literature shows that the Fe 2p peak will shift to higher energy when FePc is monodispersed on CNT or rGO surfaces due to π–π stacking,^18^ such that the Fe 2p shift in FePc/SWCNT can be attributed to the stronger interaction between the curved FePc and SWCNT. In addition, we observed experimentally a significant 1.3 eV peak splitting of N 1s for FePc/SWCNT that we attribute to the divergent nitrogen (Figure 3f), which is consistent with the theoretical calculations (Figure 1a). Figure S9 shows the ultraviolet–visible spectra (UV–vis) of the four different composites in dimethylformamide. After deposition on CNTs, a small redshift of the Q-band for FePc was observed, which we attribute to charge transfer between FePc and the CNT substrate.^32^

We further used Raman spectra to characterize the vibronic structures of our materials. As shown in Figure 3g, relative to the bare CNTs, several signals assigned to FePc vibrational peaks were observed in all FePc/CNTs, with a characteristic peak at ∼680 cm^–1^. Moreover, we observed a prominent new peak at 270 cm^–1^ for FePc/SWCNT, which was absent for FePc/MWCNT and f-FePc-SWCNT but similar to the out-of-plane deformation peak of double-walled CNT (DWCNT); we ascribe this peak to the Fe–N out-of-plane deformation, further indicating the distinct curved structure of FePc on the SWCNT surface.^22^

### Electrocatalytic ORR Performance of Catalysts

**Figure 4:**
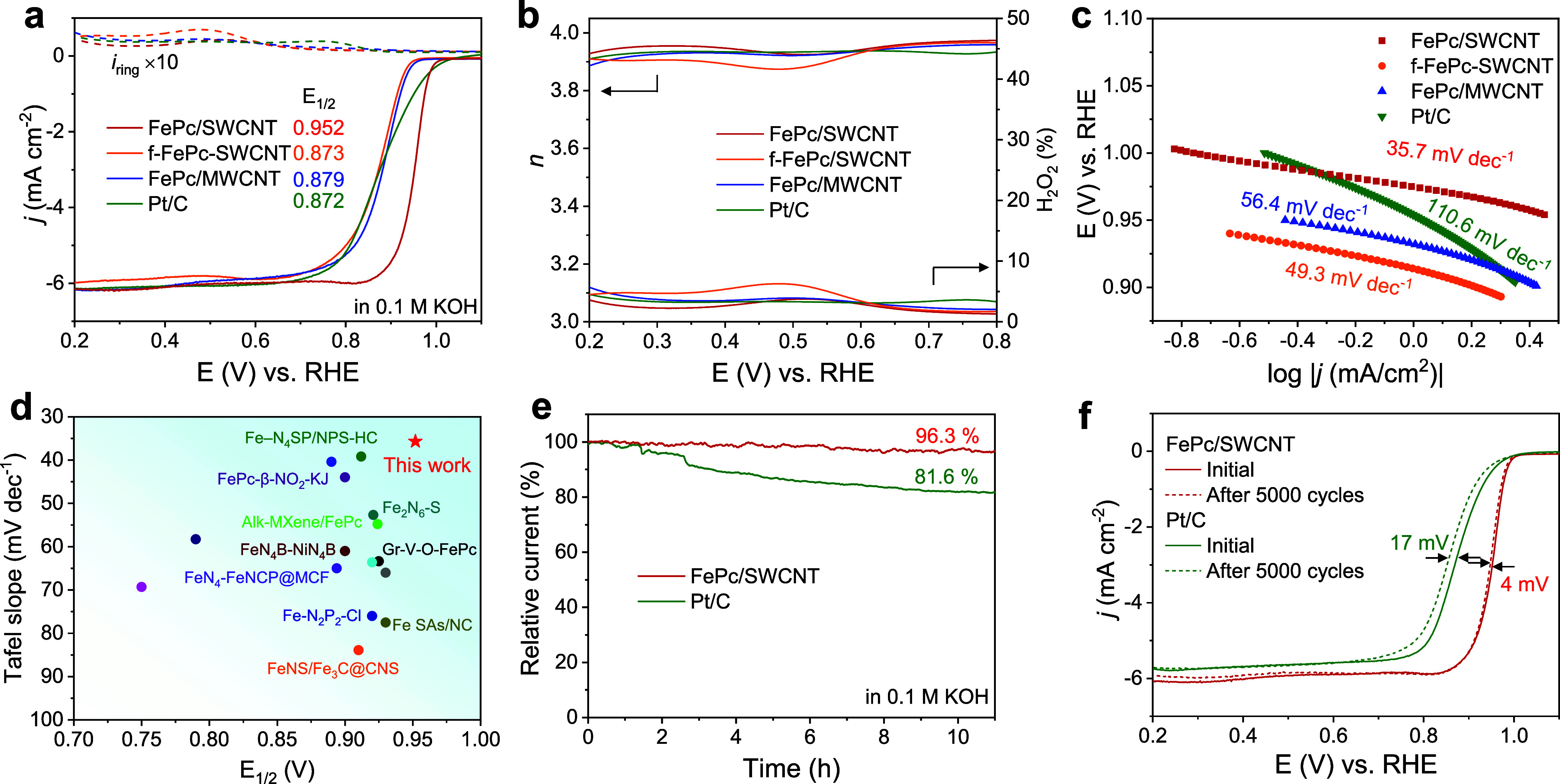
ORR performance in a 0.1 M KOH electrolyte. (a) LSV polarization curves, (b) electron transfer number and H_2_O_2_ yield, and (c) Tafel slopes of FePc/SWCNT, f-FePc-SWCNT, FePc/MWCNT, FePc, and Pt/C. (d) Comparison of the ORR performance of our FePc/SWCNT with similar reported catalysts. (e) Stability test for 11 h and (f) ORR LSV curves before and after 5000 CV cycles for FePc/SWCNT as a comparison to commercial 20% Pt/C.

The ORR electrocatalytic performance of these catalysts were assessed using a rotating disk electrode (RDE) and a rotating ring-disk electrode (RRDE) in an O_2_-saturated 0.1 M KOH electrolyte. For the Ar-saturated cyclic voltammetry (CV) curves, only two pairs of peaks could be found, which we attribute to the reduction/oxidation peaks of Fe^3+^/Fe^2+^ and Fe^2+^/Fe^+^. Under O_2_ saturation, the catalyst CV curves show obvious reduction peaks at 0.9 V, in which the FePc/SWCNT leads to the strongest reduction peak (Figure S10). As shown in Figure 4a, the linear sweep voltammetry (LSV) curves for FePc/SWCNT show the best ORR performance among all catalysts, with an *E*_1/2_ of 0.952 V, outperforming f-FePc-SWCNT (0.873 V), FePc/MWCNT (0.879 V), and Pt/C (0.872 V). This value is also higher than a recent study by Li et al., showing an *E*_1/2_ of 0.89 for strained FePc.^23^ We attributed their inferior performance to the possible formation of aggregates, as shown by the appearance of the X-ray diffraction peaks. This is also supported by our FePc aggregates on SWCNT, showing a similar *E*_1/2_ of 0.901 (Figure S2). In situ electrochemical Raman measurements were then conducted using an electrochemical Raman cell to further investigate the ORR mechanism. As shown in Figure S11a, the FePc/SWCNT catalyst shows abundant Raman scattering peaks from the molecular FePc and the D-band (1345 cm^–1^) from the SWCNT. During ORR electrocatalysis from 1 to 0 V, a new Raman signal emerged between 1080 and 1110 cm^–1^ for FePc/SWCNT. The peaks between 1000 and 1100 cm^–1^ are assigned to the O–O stretching vibration of superoxide species (O_2_^–^) absorbed at the Fe–N_4_ site.^39^ However, this O–O vibration is absent in all test potentials for FePc/MWCNT (Figure S11b). Therefore, we speculate that the strain in curved FePc promotes the formation of the *OOH intermediate during the ORR process.

LSV curves with different rotation rates and the corresponding Koutecky–Levich (K-L) plots indicate first-order reaction kinetics (Figures S12 and S13).^40^ The electron transfer number (*n*) and peroxide (H_2_O_2_) yield obtained from RRDE further confirmed the nearly four-electron O_2_ reduction pathway (Figure 4b). Specifically, in the potential range of 0.2–0.8 V, the H_2_O_2_ yield stays below 3.5% and the average *n* is calculated to be 3.96 for FePc/SWCNT. The Tafel slope for FePc/SWCNT (35.7 mV dec^–1^) is smaller than for f-FePc-SWCNT (49.3 mV dec^–1^), FePc/MWCNT (56.4 mV dec^–1^), and Pt/C (110.6 mV dec^–1^), implying faster ORR kinetics for FePc/SWCNT (Figure 4c).^41^ This marked difference in the Tafel slope indicates that the rate-determining step changes between FePc/SWCNT and FePc/MWCNT, in agreement with our theoretical calculations (Figure 1d). In comparison to recently reported Fe–N–C catalysts, our FePc/SWCNT has a smaller Tafel slope and a higher *E*_1/2_ (Figure 4d and Table S3).

To further verify the strain-induced ORR performance enhancement, we also prepared a control sample with FePc on a CNT at a diameter of ∼15 nm (FePc/15) for comparison. Figure S14 shows the ORR electrochemical performance of FePc/15 in O_2_-saturated 0.1 M KOH. The *E*_1/2_ of FePc/15 is 0.887 V (Figure S14a,c) and the electron transfer number obtained from K-L plots (Figure S14b,d) is 3.87, which are close to those of the flat FePc/MWCNT and f-FePc-SWCNT. From our simulation (Figure S15), we found that the 15 nm CNT cannot induce apparent curvature in FePc because the local curvature of the 15 nm CNT remains relatively flat to FePc, resulting in a minor change in reference to FePc/50. The electrochemical impedance spectroscopy (EIS) data of FePc/SWCNT, f-FePc-SWCNT, FePc/15, and FePc/MWCNT were also collected to compare the charge transfer resistance during ORR. The EIS data were fitted in an equivalent circuit in Figure S16, and the corresponding fitting data are shown in Table S4. The FePc/SWCNT exhibits the lowest *R*_ct_ (14.51 ohm), verifying a lower charge transfer resistance and a fast charge transfer path. These results for FePc/15 further confirm our strain-induced ORR enhancement hypothesis.

The differences in SWCNT versus MWCNT electronic interactions could also influence the charge transfer and ORR kinetics beyond geometric strain effects. Because we do not observe CNT oxidation, the accelerated electron transfer likely results from the small curvature of the SWCNT. From an orbital perspective, the strain of the CNT forces the highly delocalized π space of the CNT to localize at carbon atom centers. This localization destabilizes these π-character orbitals, increasing their energy. This increase in the orbital energy enables more facile electron transfer to FePc, translating to accelerated reduction kinetics. There are many other strategies employed for enhancing FePc ORR (Table S3). The most relevant strategies are graphitic supports or carbon nanoparticles. FePc axially bound to oxygen motifs of carbon nanoparticles was reported to achieve an *E*_1/2_ of 0.90 V.^29^ This study utilized carbon support oxidation, while we utilized support curvature. Because we achieve an *E*_1/2_ of 0.95 V, we consider our strategy to be superior.

We further explored the performance of our FePc/CNTs by evaluating their stability. Chronoamperometry at 0.7 V of FePc/SWCNT exhibits excellent long-term stability with 96.3% current retention after 11 h, while the Pt/C current decreases to 81.6% (Figure 4e). Raman (Figure S17) and UV–vis (Figure S18) characterization of the FePc/SWCNT after the stability test confirms that the curved FePc structure is well-maintained. The excellent durability of FePc/SWCNT is also confirmed by the accelerated durability test (ADT; Figure 4f). After 5000 CV cycles from 0.6 to 1.0 V, the *E*_1/2_ of FePc/SWCNT only decreases by 4 mV, significantly lower than for Pt/C (17 mV). We attribute the long-term stability to the lack of CNT oxidation and Fe leaching enabled by the formal reduction. In addition, the ORR working potential is higher than −0.4 V, thus avoiding Fe(0) formation.^42^ Meanwhile, FePc/SWCNT also presents a stronger resistance to methanol poisoning than Pt/C (Figure S19).

### Performance of Zinc–Air Batteries

**Figure 5:**
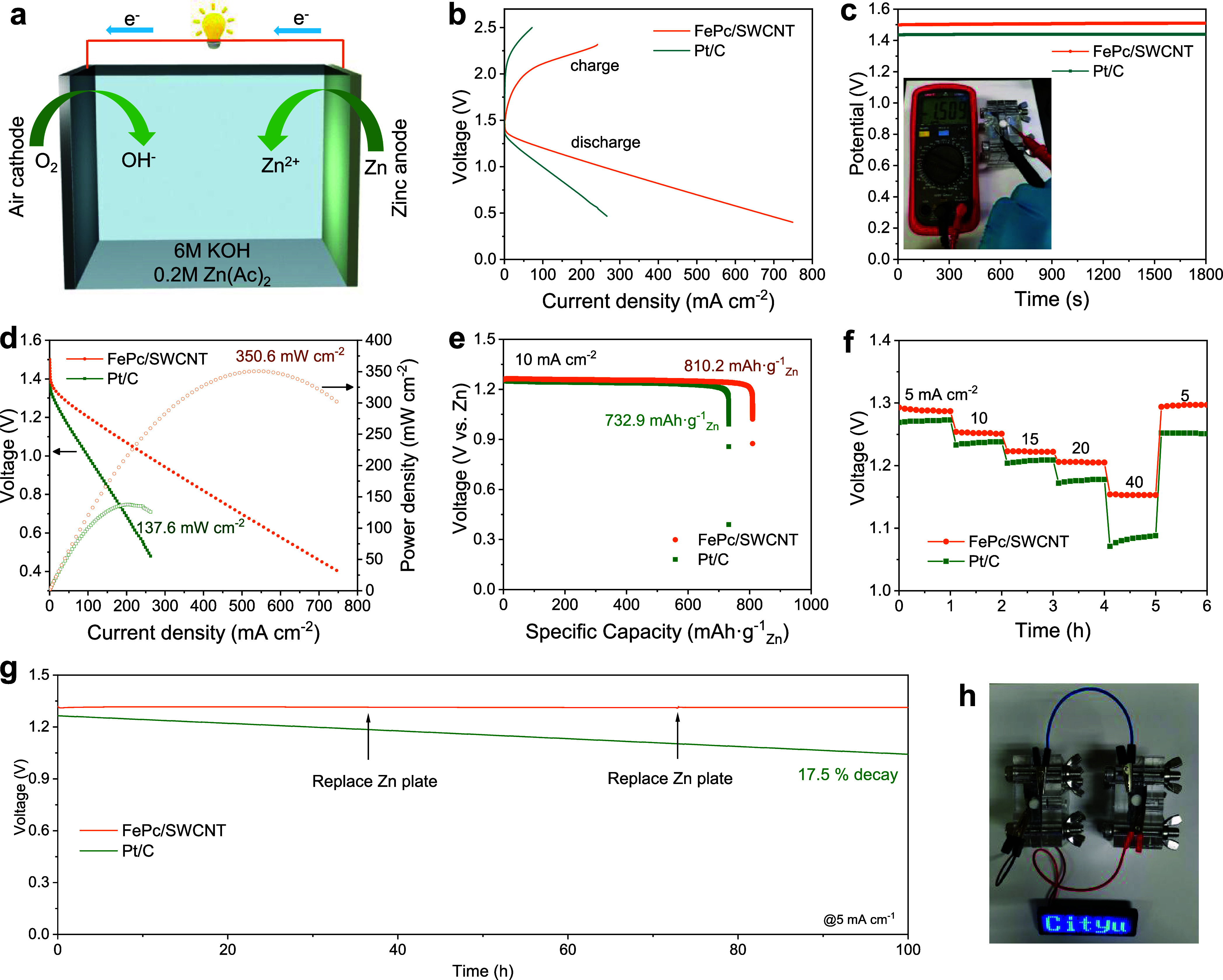
Electrochemical performance of the zinc–air battery. (a) Schematic structure of ZAB. (b) Charging and discharging polarization curves and (c) OCV curves of FePc/SWCNT- and Pt/C-based ZABs. Inset: optical picture of the FePc/SWCNT-based ZAB with an OCV of 1.509 V. (d) Discharge polarization and power density curves, (e) specific capacity curves at 10 mA cm^–2^, (f) discharge curves at different discharge current densities from 5 to 40 mA cm^–2^ of FePc/SWCNT as a comparison to 20% Pt/C. (g) Durability test of the FePc/SWCNT- and Pt/C-based ZAB for 100 h at 5 mA cm^–2^. (h) Illustration of the optical image of an LED panel lit by two ZABs in series.

We finally showcase the superior ORR performance of the FePc/SWCNT catalyst in a homemade aqueous Zn–air battery (ZAB). This battery uses FePc/SWCNT as the air cathode, a Zn plate as the anode, and 6.0 M KOH with 0.2 M Zn(CH_3_COO)_2_ as the electrolyte (Figure 5a). We also investigated commercial Pt/C as the air cathode for comparison. As shown in Figure 5b, FePc/SWCNT-ZAB exhibited a 0.5 V smaller voltage gap compared with Pt/C, implying higher energy efficiency. In Figure 5c, FePc/SWCNT-ZAB displayed a higher open-circuit voltage (OCV) of 1.51 V than the Pt/C-based battery (1.44 V). The FePc/SWCNT-ZAB also delivered a maximum peak power density of 350.6 mW cm^–2^ (Figure 5d), which is 2.55 times higher than that of Pt/C (137.6 mW cm^–2^). This FePc/SWCNT-ZAB performance is also superior to many recently reported results, which are typically in the range of 150–300 mW cm^–2^ (Table S5), including some recently reported Fe-based catalysts.^43,44^ The discharge-specific capacity (Figure 5e) of FePc/SWCNT-ZAB was 810.2 mA h g_Zn_^–1^ at 10 mA cm^–2^, which exceeds that of Pt/C (732.9 mA h g_Zn_^–1^). We also found that the voltage variation among the FePc/SWCNT-ZAB discharge curves is much smaller than that for Pt/C under various current densities, revealing its excellent rate performance and reversibility (Figure 5f). Moreover, our FePc/SWCNT-ZAB demonstrates excellent stability for 100 h with negligible voltage attenuation at 5 mA cm^–2^ (Figure 5g), while Pt/C-ZAB decays by 17.5% within 100 h. As shown in Figure 5h, two FePc/SWCNT-ZABs connected in series can power a light-emitting diode display.

## Conclusions

The present study reveals that monodispersed FePc on single-walled carbon nanotubes expedites electron transfer, giving rise to accelerated oxygen reduction relative to FePc on multi-walled carbon nanotubes. For flat FePc-C_24_, the conversion of O_2_ (g) to *OOH is rate-limiting at 1.0 V with a free energy change of 0.33 eV. For curved FePc-C_24_, *OH → OH^–^ is rate-limiting with a free energy change of 0.27 eV. This observed ΔΔ*G* of 0.06 eV aligns with the experimentally observed enhancement in ORR for FePc/SWCNT over FePc/MWCNT (*E*_1/2_ = 0.952 and 0.879 V for FePc/SWCNT and FePc/MWCNT, respectively). Moreover, the change in the rate-limiting step (O_2_ (g) → *OOH for flat and *OH → OH^–^ for curved) is supported by the change in the experimental Tafel slope (35.7 and 56.4 mV dec^–1^ for FePc/SWCNT and FePc/MWCNT, respectively). Experiments reveal that the strained FePc/SWCNT catalyzes ORR with remarkable speed, outperforming that of conventional Fe–N–C materials.

Using the FePc/SWCNT as the air electrode, the as-assembled zinc–air battery exhibits superior performance with an open-circuit voltage of 1.51 V and a maximum peak power density of 350.6 mW cm^–2^. Our study suggests that molecular strain changes the adsorption of key intermediates while simultaneously accelerating electron transfer for key reduction steps, thereby providing a promising approach for enhanced catalytic activity.

## Supporting Information Available

Additional experimental procedures and materials characterizations including methods, images of catalysts, spectroscopic data, and performance data (DOCX)
