# Supplementary material for: Molecular Strain Accelerates Electron Transfer for Enhanced Oxygen Reduction
Source: J Am Chem Soc. 2025 Jan 17;147(4):3786–95. doi: 10.1021/jacs.4c16637 (PMC11783534; doi:10.1021/jacs.4c16637)
Supplement: Supplementary file 1 — ja4c16637_si_001.docx [file ja4c16637_si_001.docx]

Supplemental information

**Molecular Strain Accelerates Electron Transfer for Enhanced Oxygen Reduction**

Charles B. Musgrave III,^a,#^ Jianjun Su,^b,#^ Pei Xiong,^c^ Yun Song,^b^ Libei Huang,^b,d^ Yong Liu,^b^ Geng Li,^b^ Qiang Zhang,^b^ Yinger Xin,^b^ Molly Meng-Jung Li,^c^ Ryan Tsz Kin Kwok,^e^ Jacky W. Y. Lam,^e^ Ben Zhong Tang,^e,f^ William A. Goddard III,^a*^ Ruquan Ye^b,g*^

*^a^Materials and Process Simulation Center, California Institute of Technology, Pasadena, CA, 91125, USA*

*^b^Department of Chemistry, State Key Laboratory of Marine Pollution, City University of Hong Kong, Hong Kong, China.*

*^c^Department of Applied Physics, Hong Kong Polytechnic University, Hong Kong.*

*^d^Division of Science, Engineering and Health Study, School of Professional Education and Executive Development (PolyU SPEED), The Hong Kong Polytechnic University, Hong Kong 999077, P. R. China.*

*^e^Department of Chemistry and the Hong Kong Branch of Chinese National Engineering Research Center for Tissue Restoration and Reconstruction, The Hong Kong University of Science and Technology, Hong Kong 999077, China.*

*^f^School of Science and Engineering, Shenzhen Institute of Aggregate Science and Technology, The Chinese University of Hong Kong, Shenzhen, Guangdong 518172, China.*

*^g^City University of Hong Kong Shenzhen Research Institute, Shenzhen, Guangdong 518057, China*

**Correspondence: wag@caltech.edu; ruquanye@cityu.edu.hk*

**Experimental Procedures**

**Synthesis of catalysts**

The SWCNT and MWCNT (with a diameter of 50 nm) were bought from XFNANO. Before use, CNT samples were annealed at 300 ^o^C in air for 2 h and then immersed in 6 mol L^−1^ HCl solution for 12 h to remove metallic species. The annealing process is important to remove some surface amorphous carbon (Figure S1), which might interfere with the molecule-CNT interactions. After washing with deionized water, the samples were freeze-dried. 20 mg of the purified SWCNT were dispersed in 20 ml of DMF with ultrasonic treatment for 30 min, followed by adding the appropriate amount of iron (II) phthalocyanine (J&K Scientific Ltd). The mixture was sonicated for 30 min to obtain a well-mixed suspension, which was further stirred at room temperature for 24 h. Subsequently, the mixture was centrifuged and successively washed with DMF, ethanol and deionized water several times. Finally, the precipitates were freeze-dried overnight, denoted as FePc/SWCNT. The FePc/MWCNT was prepared using the same processes, except that MWCNT was used instead of SWCNT. We also prepared a semi-curved FePc on CNT with ~15 nm diameter (FePc/15) for comparison. The f-FePc-SWCNT was synthesized through a modified covalently grafting strategy^1^. The weight percentage of Fe in all samples is ~0.28% determined by ICP.

**Materials characterization**

The morphology of samples was characterized using transmission electron microscopy (TEM, Philips Technai 12) equipped with energy dispersive X-ray spectroscopy. ICP-atomic emission spectroscopy (ICP-OED) measurements were conducted on Optima 8000 spectrometer. Samples were digested in hot concentrated HNO_3_ for 1 h and diluted to desired concentrations. UV-vis spectrum was performed on a Shimadzu 1700 spectrophotometer in DMF solution. The X-ray photoelectron spectroscopy data were collected on a Thermo ESCALAB 250Xi spectrometer equipped with a monochromatic AlK radiation source (1486.6 eV, pass energy 20.0 eV). The data were calibrated with C 1s 284.6 eV. Raman spectra were collected using a LabRAM HR800 laser confocal micro-Raman spectrometer with a laser wavelength of 514.5 nm.

X-ray absorption fine spectroscopy (XAFS) measurements were performed in the fluorescence mode using a Lytle detector at beamline 01C1 of National Synchrotron Radiation Research Center (NSRRC) in Taiwan. The electron storage ring was operated at 1.5 GeV with a constant current of ~ 360 mA. A Si (111) Double Crystal Monochromator (DCM) was used to scan the photon energy. XANES analyses were conducted using the Athena software based on the IFEFFIT program^2^ to determine the structural environment of Fe atoms. Averaged XAS spectra were first normalized to the absorption edge height, and the background was removed using the automatic background subtraction routine AUTOBK implemented in the Athena software^3^. A reference foil of Fe foil was used for energy calibration of the monochromator, which was applied to all spectra. Quantitative information on the radial distribution of neighboring atoms surrounding Fe atoms was derived from the extended absorption fine structure (EXAFS) data. An established data reduction method was used to extract the EXAFS χ-functions from the raw experimental data using the IFEFFIT software.

**Electrochemical measurements**

RDE tests were performed in O_2_ saturated 0.1 mol L^-1^ KOH solution with a scan rate of 10 mV s^-1^ between 1.1 V and 0.2 V at different rotating rates using PINE 636 rotating-disk electrode system and CHI 760E workstation. Ag/AgCl, Pt and glassy carbon were used as reference, counter and working electrodes, respectively. Catalyst ink was prepared by ultrasonic treatment of the mixture of 2 mg catalyst, 980 μL ethanol and 20 μL of 5 wt.% Nafion solution (Sigma Aldrich) for 1h. O_2_ was saturated in the electrolyte for 30 min before the tests. All potentials were converted to the reversible hydrogen electrode (RHE) according to the Nernst equation (E (versus RHE) = E (versus Ag/AgCl) + 0.23 + 0.0592 × pH).

The electron transfer number (n) was determined by the Koutecky-Levich Equation (1-2):

$\frac{1}{J}=\frac{1}{J_{L}}+\frac{1}{J_{K}}=\frac{1}{B\omega^{\frac{1}{2}}}+\frac{1}{J_{K}}$ (1)

$B=0.62nFC_{0}D_{0}^{\frac{2}{3}}V^{-\frac{1}{6}}$ (2)

where *J* is the measured current density, *J_K_* and *J_L_* are the kinetic and limiting current densities, *ω* is the angular velocity of the disk, *n* is the overall number of electrons transferred in oxygen reduction, F is the Faraday constant (96485 C mol^-1^), C_0_ is the bulk concentration of O_2_ (1.2 × 10^-6^ mol cm^-3^), D_0_ is the diffusion coefficient of O_2_ in 0.1 M KOH (1.9 × 10^-5^ cm^2^ s^-1^), and V is the kinematic viscosity of the electrolyte (0.01 cm^2^ s^-1^).

The electron transfer number (*n*) and peroxide yield (%H_2_O_2_) were calculated from the ring current (*I_ring_*) and the disk current (*I_disk_*) through the following equations:

$n=4\times\frac{I_{disk}}{I_{disk+}I_{ring/N}}$ (3)

$H_{2}O_{2}\%=200\times\frac{I_{ring}/N}{I_{disk+}I_{ring/N}}$ (4)

where *I_disk_* is the faradaic current at the disk, *I_ring_* is the faradaic current at the ring, and N is the H_2_O_2_ collection coefficient at the ring (N=0.37).

The electrochemical impedance spectra were recorded with an AC amplitude of 5 mV by sweeping the frequency from 100 kHz to∼1 Hz at half-wave potential in O_2_-satureted 0.1 M KOH solution. The parameters were fitted using ZView software. In situ Raman measurements were conducted using the electrochemical Raman cell on a conformal Raman microscope (Renishaw, inViaTM) with an excitation wavelength of 532 nm. The working electrode was prepared by dropping the catalyst ink onto the carbon paper. Each potential dependent Raman spectrum was acquired over a collection time of 15 s after holding at each potential for 10 minutes between 0 V and 1.0 V vs. RHE. The methanol tolerance was tested by chronoamperometry at 0.7 V vs. RHE in an O_2_-saturated system with the injection of 2M methanol at an RDE rotation rate of 1600 rpm. The accelerated deterioration tests (ADT) were examined by 5000 CV cycles between 0.6 and 1.0 V (vs. RHE).

**Assembly of zinc-air batteries**

The aqueous ZAB were evaluated using a homemade instrument under ambient atmospheric conditions. A polished Zn plate as the anode, a catalyst-coated gas diffusion layer (1mg cm^-2^ catalyst loading) as the air cathode and the mixed solution of 6 M KOH and 0.2 M Zn(CH_3_COO)_2_ as the electrolyte. The electrochemical tests of ZAB were conducted on an electrochemical workstation (CHI760e) in a two-electrode system under ambient air. The charge and discharge polarization curves were collected at a scan rate of 10 mV s^-1^. The specific discharge capacity was normalized to the mass of the consumed Zn plate based on galvanostatic discharge results.

**Computational methods**

Periodic Density Functional Theory (DFT) calculations were performed using the Vienna Ab initio Simulation Package (VASP) version 6.3.2^4^. Optimizations were initially carried out using the PBE generalized gradient approximation (GGA) functional,^5^ including the Grimme D3BJ empirical correction for London Dispersion interactions.^6^ All optimizations featured Projector Augmented Wave (PAW) pseudopotentials for all atoms.^7^ The plane wave basis set cutoff was set to 500 eV, and a 1 x 1 x 1 K point grid was used. We also used VASP to calculate phonons via the finite difference method. Phonons at 298.15 K were used to predict free energies at 298.15 K, including zero-point energy and entropy. A Hubbard U correction of 5 eV was used for Fe to account for correlated d electrons that are not properly described by simple GGA functionals like PBE.^8^

VASP geometry minimizations were followed by solution-phase single point energy calculations, which were carried out using the JDFTx software package to obtain the energetics as a function of applied potential.^9^ The JDFTx solvent calculations included implicit water solvation via the CANDLE linear solvation model.^10^ Coulomb interactions were truncated in all 3 cartesian directions to avoid artificial interactions of the FePc with itself in adjacent periodic images.

Hybrid DFT calculations were performed using the ORCA v5.0.4 quantum chemistry software.^11^ Structural optimizations were first performed with the B3LYP functional^12^ augmented with the D3BJ empirical correction for London dispersion forces. Following B3LYP structural optimization, single-point energy calculations were performed with the Head-Gordon ⍵B97M-V range-separated functional,^13^ which includes VV10 nonlocal correlation.^14^ In all calculations, the def2-TZVP basis set was used.^15^ All calculations also featured implicit water solvent via the ORCA Conductor-like Polarizable Continuum Model (CPCM).^16^

To capture how the reaction free energy surface changes with applied potential, we also computed Grand Canonical Potential (GCP) free energies.^17^ The GCP is computed by taking the Legendre transform of Gibbs free energy with respect to the number of electrons (Ne), such that the conjugate variable that appears is the chemical potential, $\mu$.^18^ For GCP to serve as a thermodynamic potential, Ne must be equilibrated to match the desired applied voltage. This is achieved by self-consistently varying Ne in order to match the fermi energy to the applied voltage, as in Equation (5). The Computational Hydrogen Electrode (CHE) method is used for the hydrogen reference, in which the potential of a proton-electron pair is equivalent to the free energy of ½ H_2_ at standard conditions.

$\mu=\frac{dG(N_{e})}{N_{e}}=e(U_{SHE}-U)$ (5)

**Figure S1.** TEM images of (a) unannealed SWCNT and (b) ORR LSV curves of FePc/SWCNT with annealed and unannealed SWCNT samples.

**Figure S2.** (a) Raman spectrum and (b) ORR performance of FePc/SWCNT with different molecular/SWCNT ratios.

**Figure S3.** SEM images of FePc/SWCNT with different molecular/SWCNT mass ratio: (a) 1:10, (b) 2:10, (c) 4:10 and (d) 8:10.


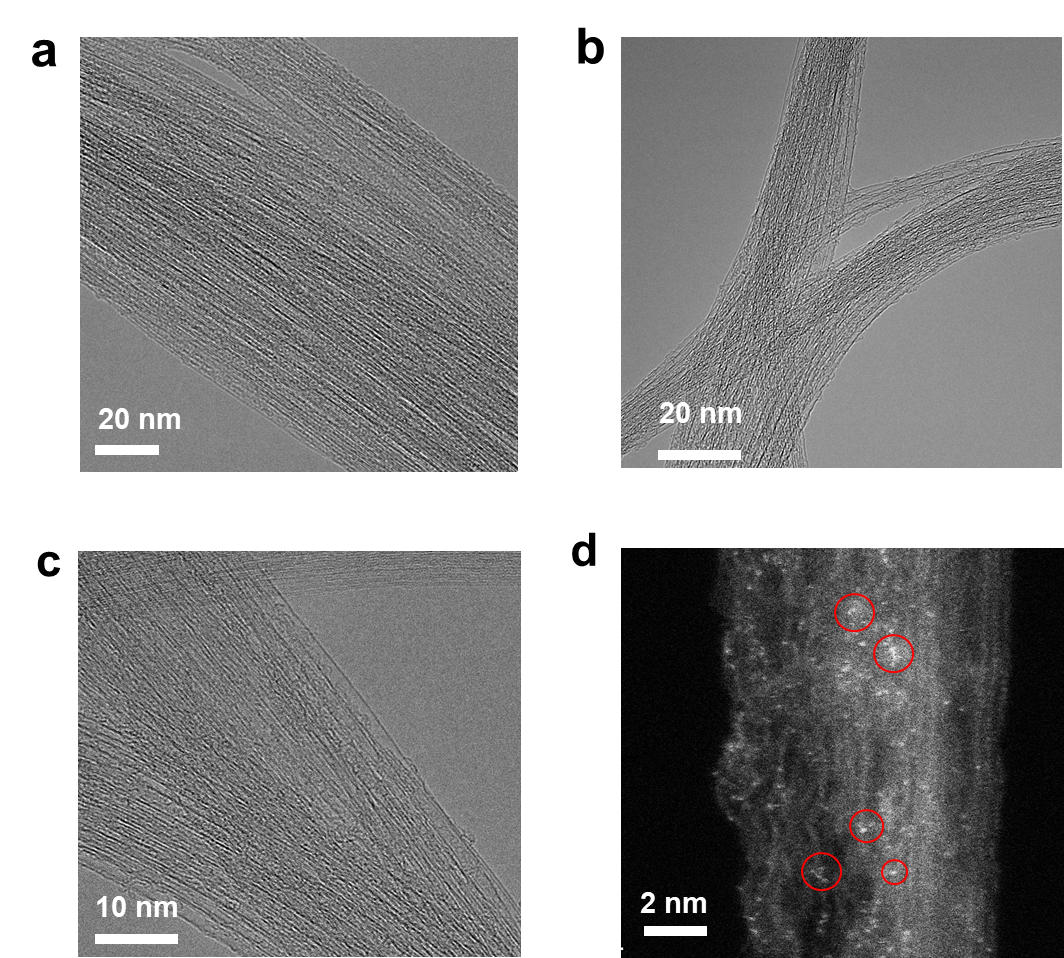


**Figure S4.** TEM images of (a) SWCNT, (b) f-FePc-SWCNT, (c) FePc/SWCNT. (d) HAADF-STEM image of FePc/SWCNT.


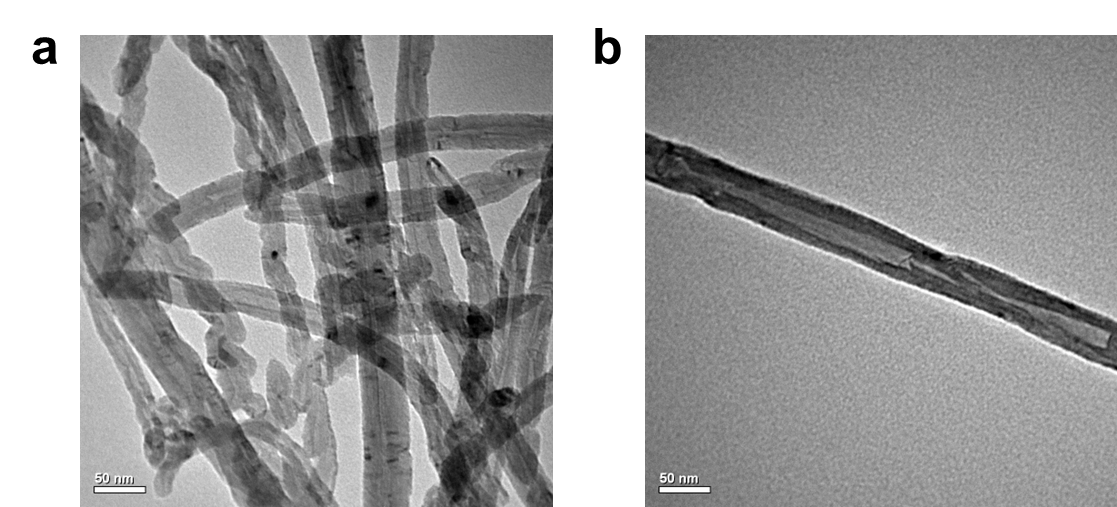


**Figure S5.** TEM images of (a) MWCNT and (b) FePc/MWCNT. The diameter of the MWCNT is approximately 50 nm.


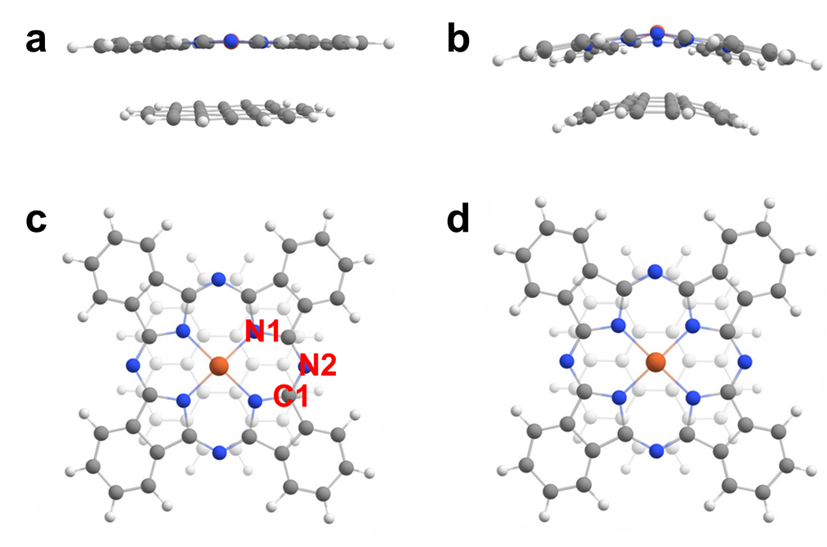


**Figure S6.** Simulated structures of (a) side and (c) top view for FePc/MWCNT and (b) side and (d) top view for FePc/SWCNT.

**Figure S7.** EXAFS fitting at k space of (a) FePc and (b) FePc/SWCNT. EXAFS fitting at R space of (c) FePc and (d) FePc/SWCNT.

**Figure S8.** EXAFS fitting at k space of (a) f-FePc-SWCNT and (b) FePc/MWCNT. EXAFS fitting at R space of (c) f-FePc-SWCNT and (d) FePc/MWCNT.

**Figure S9.** UV-vis of FePc/SWCNT, f-FePc-SWCNT, FePc/MWCNT and FePc.

**Figure S10.** CV curves of (a) FePc/SWCNT, (b) FePc/MWCNT, (c) f-FePc-SWCNT and (d) Pt/C in Ar- and O_2_-saturated 0.1 M KOH solution.

**Figure S11.** In-situ Raman spectra of the (a) FePc/SWCNT and (b) FePc/MWCNT from 1.0 V to 0 V in O_2_-saturated 0.1 M KOH solution.

**Figure S12.** (a) and (c) LSV curves of FePc/SWCNT and (b) FePc/MWCNT at different rotation speeds. (b) and (d) are the corresponding K-L plots.

**Figure S13.** (a) and (c) LSV curves of f-FePc-SWCNT and (b) Pt/C at different rotation speeds. (b) and (d) are the corresponding K-L plots.

**Figure S14.** ORR performance of FePc/15 in O_2_-saturated 0.1M KOH. (a) LSV curves at different rotation speeds. (b) Corresponding K-L plots. (c) LSV polarization curve at 1600 rpm and (d) Electron transfer number and H_2_O_2_ yield.

**Figure S15.** Simulated structures of FePc/15: (a) side view and (b) top view.

**Figure S16.** Nyquist plots of EIS of FePc/SWCNT, FePc/15, f-FePc-SWCNT and FePc/MWCNT at half-wave potential in O_2_-satureted 0.1 M KOH solution. The symbols and solid lines show the experimental and the fitted data, respectively.

**Figure S17.** Raman spectrum of FePc/SWCNT after stability test.

**Figure S18.** UV-vis spectrum of FePc/SWCNT after stability test.

**Figure S19.** Methanol tolerance tests of FePc/SWCNT and Pt/C at 0.7 V vs.RHE with an addition of 2.0 M methanol.

**Table S1.** Summary of Fe K-edge EXAFS fitting results of FePc/SWCNT, f-FePc-SWCNT, FePc/MWCNT and FePc.

| **Sample** | **Path** | **EXAFS** | | **D-W factor (σ^2^),** Å^2^ | **Enot, eV** | **R-factor, %** |
| --- | --- | --- | --- | --- | --- | --- |
|  |  | CN | R, Å |  |  |  |
| FePc | Fe-N1 | 4.2 (2) | 1.969 (8) | 0.005 (1) | 6.9 (7) | 1.5 |
|  | Fe-C1 | 7.7 (8) | 2.987 (1) | 0.003 (1) | 6.8 (9) |  |
|  | Fe-N2 | 4.1 (9) | 3.272 (2) | 0.005 (3) | 6.9 (7) |  |
| FePc/MWCNT | Fe-N1 | 4.2 (5) | 1.975 (6) | 0.003 (1) | 5.5 (4) | 1.9 |
|  | Fe-C1 | 8.0 (7) | 2.992 (5) | 0.007 (3) | 4.6 (3) |  |
|  | Fe-N2 | 4.0 (4) | 3.262 (3) | 0.011 (1) | 5.5 (4) |  |
| FePc/SWCNT | Fe-N1 | 4.0 (1) | 1.998 (3) | 0.005 (6) | 7.9 (5) | 0.3 |
|  | Fe-C1 | 8.0 (6) | 2.998 (5) | 0.013 (5) | 8.8 (6) |  |
|  | Fe-N2 | 3.9 (3) | 3.250 (7) | 0.014 (2) | 7.9 (5) |  |
| f-FePc-SWCNT | Fe-N1 | 3.4 (4) | 1.974 (5) | 0.003 (2) | 8.7 (6) | 0.5 |
|  | Fe-C1 | 6.9 (5) | 2.924 (6) | 0.003 (5) | 9.3 (5) |  |
|  | Fe-N2 | 3.9 (7) | 3.254 (5) | 0.006 (3) | 8.7 (6) |  |

**Table S2.** Summary of geometry change for curved FePc/SWCNT and flat FePc/MWCNT from DFT simulations

|  | FePc/SWCNT | FePc/MWCNT |
| --- | --- | --- |
| Curvature (^o^) | 12.8 | 0.44 |
| Fe-N_1(1)_ (Å) | 1.950 | 1.955 |
| Fe-N_1(2)_ (Å) | 1.983 | 1.957 |
| Fe-N_1(3)_ (Å) | 1.950 | 1.955 |
| Fe-N_1(4)_ (Å) | 1.984 | 1.956 |

**Table S3.** Comparison of the ORR performance for FePc/SWCNT with reported similar catalysts in 0.1 M KOH solution.

| Name | E_1/2_  (V vs RHE) | Tafel slope  (mV dec^-1^) | Ref. |
| --- | --- | --- | --- |
| FePc/SWCNT | 0.952 | 35.7 | This work |
| Gr-V-O-FePc | 0.925 | 63.37 | 19 |
| Alk-MXene/FePc | 0.924 | 54.79 | 20 |
| S-Zn-N-C-950 | 0.89 | 40.4 | 21 |
| Cu-Co/NC | 0.92 | 63.6 | 22 |
| CuCo_2_O_4-x_S_x_/NC-2 | 0.75 | 69.3 | 23 |
| Fe SAs/NC | 0.93 | 77.5 | 24 |
| Co-PorBpy-Co | 0.79 | 58.3 | 25 |
| FePc-β-NO_2_-KJ | 0.9 | 44 | 26 |
| FeN_4_B-NiN_4_B | 0.9 | 61 | 27 |
| Fe–N_4_SP/NPS-HC | 0.912 | 39.18 | 28 |
| Fe_2_N_6_-S | 0.921 | 52.7 | 29 |
| Fe-N_2_P_2_-Cl | 0.92 | 76 | 30 |
| FeNS/Fe_3_C@CNS | 0.91 | 83.9 | 31 |
| FeN_4_-FeNCP@MCF | 0.894 | 65 | 32 |
| CR-Co/ClNC | 0.93 | 66 | 33 |

**Table S4.** The equivalent circuits fitting results of FePc/SWCNT, FePc/15, f-FePc-SWCNT and FePc/MWCNT in 0.1 M KOH.

| **Sample** | **Rs (Ω)** | **Error (%)** | **C (μF)** | **Error (%)** | **Rct (Ω)** | **Error (%)** |
| --- | --- | --- | --- | --- | --- | --- |
| FePc/SWCNT | 2.104 | 0.44 | 1.934 | 0.36 | 14.51 | 0.26 |
| f-FePc-SWCNT | 2.411 | 0.46 | 1.724 | 0.38 | 16.50 | 0.30 |
| FePc/15 | 2.370 | 0.79 | 1.724 | 0.65 | 16.07 | 0.64 |
| FePc/MWCNT | 2.330 | 0.74 | 1.470 | 0.56 | 15.77 | 0.57 |

**Table S5.** Comparison of the performance of Zn-air batteries reported in recent literature.

| Air-cathode | Open-circuit voltage (V) | Peak power  density (mW  cm^-2^) | Specific capacity  (mAh g^-1^) | Ref. |
| --- | --- | --- | --- | --- |
| FePc/SWCNT | 1.51 | 350.6 | 810.2 | This work |
| Gr-V-O-FePc | 1.44 | 167 | 804.6 | 19 |
| Alk-MXene/FePc | 1.487 | 191.5 | 820 | 20 |
| S-Zn-N-C-950 | 1.42 | 229.2 | 817 | 21 |
| Cu-Co/NC | 1.45 | 295.9 | 752.2 | 22 |
| Fe SAs/NC | 1.46 | 306.1 | 785.4 | 24 |
| FeN_4_B-NiN_4_B | 1.498 | 236.9 | 771.7 | 27 |
| Fe-N_2_P_2_-Cl | 1.495 | 260 | 812 | 30 |
| CR-Co/ClNC | 1.50 | 176.6 | 745 | 33 |
| FePc/DG | 1.29 | 190 | 735 | 34 |
| Fe_SAs+NPs_Ce_SAs+Fe-ONPs_/NC | 1.55 | 240.5 | 788.2 | 35 |
| FeS/FeNSC | 1.50 | 256.06 | 807.5 | 36 |
| FeCoN_5_C/SL | 1.483 | 224.8 | 803 | 37 |
| FePc-NHCS-500 | 1.524 | 230 | 808.7 | 38 |
| Fe–N,O/G | 1.55 | 164.7 | - | 39 |
| CoFeCu-TAC | 1.538 | 208 | 806.45 | 40 |
| NiFe–N–C | 1.59 | 153 | 818 | 41 |
| Fe_sa_Cu_nc_/NC | - | 234.7 | 767.6 | 42 |
| FeNC-VN | 1.55 | 299.4 | 798 | 43 |
|  |  |  |  |  |

**REFERENCES**

(1) Su, J.; Zhang, J. J.; Chen, J.; Song, Y.; Huang, L.; Zhu, M.; Yakobson, B. I.; Tang, B. Z.; Ye, R. Building a Stable Cationic Molecule/Electrode Interface for Highly Efficient and Durable CO_2_ Reduction at an Industrially Relevant Current. *Energy Environ. Sci.* **2021**, *14*, 483–492.

(2) Ravel, B.; Newville, M. ATHENA, ARTEMIS, HEPHAESTUS: Data Analysis for X-Ray Absorption Spectroscopy Using IFEFFIT. *J. Synchrotron Radiat.* **2005**, *12*, 537–541.

(3) Newville, M. IFEFFIT : Interactive XAFS Analysis and FEFF Fitting. *J. Synchrotron Radiat.* **2001**, *8*, 322–324.

(4) Arias, M. M. Vienna Ab-Initio Simulation Package. *Univ. Edinburgh Edinburgh, UK* **2009**.

(5) Perdew, J. P.; Burke, K.; Ernzerhof, M. Generalized Gradient Approximation Made Simple. *Phys. Rev. Lett.* **1996**, *77*, 3865–3868.

(6) Grimme, S.; Antony, J.; Ehrlich, S.; Krieg, H. A Consistent and Accurate Ab Initio Parametrization of Density Functional Dispersion Correction (DFT-D) for the 94 Elements H-Pu. *J. Chem. Phys.* **2010**, *132*, 154104.

(7) Kresse, G.; Joubert, D. From Ultrasoft Pseudopotentials to the Projector Augmented-Wave Method. *Phys. Rev. B* **1999**, *59*, 1758–1775.

(8) Brena, B.; Puglia, C.; De Simone, M.; Coreno, M.; Tarafder, K.; Feyer, V.; Banerjee, R.; Göthelid, E.; Sanyal, B.; Oppeneer, P. M.; Eriksson, O. Valence-Band Electronic Structure of Iron Phthalocyanine: An Experimental and Theoretical Photoelectron Spectroscopy Study. *J. Chem. Phys.* **2011**, *134*, 074312.

(9) Sundararaman, R.; Letchworth-Weaver, K.; Schwarz, K. A.; Gunceler, D.; Ozhabes, Y.; Arias, T. A. JDFTx: Software for Joint Density-Functional Theory. *SoftwareX* **2017**, *6*, 278–284.

(10) Sundararaman, R.; Goddard, W. A. The Charge-Asymmetric Nonlocally Determined Local-Electric (CANDLE) Solvation Model. *J. Chem. Phys.* **2015**, *142*, 064107.

(11) Neese, F.; Wennmohs, F.; Becker, U.; Riplinger, C. The ORCA Quantum Chemistry Program Package. *J. Chem. Phys.* **2020**, *152*, 224108.

(12) Beeke, A. D. Density-Functional Thermochemistry. III. The Role of Exact Exchange. *J. Chem. Phys* **1993**, *98*, 5646–5648.

(13) Mardirossian, N.; Head-Gordon, M. ω B97M-V: A Combinatorially Optimized, Range-Separated Hybrid, Meta-GGA Density Functional with VV10 Nonlocal Correlation. *J. Chem. Phys.* **2016**, *144*, 214110.

(14) Vydrov, O. A.; Van Voorhis, T. Nonlocal van Der Waals Density Functional: The Simpler the Better. *J. Chem. Phys.* **2010**, *133*, 244103.

(15) Weigend, F.; Ahlrichs, R. Balanced Basis Sets of Split Valence, Triple Zeta Valence and Quadruple Zeta Valence Quality for H to Rn: Design and Assessment of Accuracy. *Phys. Chem. Chem. Phys.* **2005**, *7*, 3297.

(16) Barone, V.; Cossi, M. Conductor Solvent Model. *J. Phys. Chem. A* **1998**, *102* , 1995–2001.

(17) Sundararaman, R.; Goddard, W. A.; Arias, T. A. Grand Canonical Electronic Density-Functional Theory: Algorithms and Applications to Electrochemistry. *J. Chem. Phys.* **2017**, *146*, 114104.

(18) Huang, Y.; Nielsen, R. J.; Goddard, W. A. Reaction Mechanism for the Hydrogen Evolution Reaction on the Basal Plane Sulfur Vacancy Site of MoS 2 Using Grand Canonical Potential Kinetics. *J. Am. Chem. Soc.* **2018**, *140*, 16773–16782.

(19) Li, X.; Wu, X.; Zhao, Y.; Lin, Y.; Zhao, J.; Wu, C.; Liu, H.; Shan, L.; Yang, L.; Song, L.; Jiang, J. Promoting Oxygen Reduction Reaction by Inducing Out‐of‐Plane Polarization in a Metal Phthalocyanine Catalyst. *Adv. Mater.* **2023**, *35*, 2302467.

(20) Dai, Y.; Liu, B.; Zhang, Z.; Guo, P.; Liu, C.; Zhang, Y.; Zhao, L.; Wang, Z. Tailoring the D‐Orbital Splitting Manner of Single Atomic Sites for Enhanced Oxygen Reduction. *Adv. Mater.* **2023**, *35*, 2210757.

(21) Tan, Y.; Zhang, Z.; Chen, S.; Wu, W.; Yu, L.; Chen, R.; Guo, F.; Wang, Z.; Cheng, N. Local Geometric Distortion to Stimulate Oxygen Reduction Activity of Atomically Dispersed Zn-Nx Sites for Zn–Air Batteries. *Adv. Funct. Mater.* **2023**, *34*, 2311337.

(22) Li, Z.; Ji, S.; Wang, C.; Liu, H.; Leng, L.; Du, L.; Gao, J.; Qiao, M.; Horton, J. H.; Wang, Y. Geometric and Electronic Engineering of Atomically Dispersed Copper‐Cobalt Diatomic Sites for Synergistic Promotion of Bifunctional Oxygen Electrocatalysis in Zinc–Air Batteries. *Adv. Mater.* **2023**, *35*, 2300905.

(23) Cai, J.; Zhang, H.; Zhang, L.; Xiong, Y.; Ouyang, T.; Liu, Z. Q. Hetero-Anionic Structure Activated Co-S Bonds Promote Oxygen Electrocatalytic Activity for High-Efficiency Zinc–Air Batteries. *Adv. Mater.* **2023**, *35*, 2303488.

(24) Li, Z.; Ji, S.; Xu, C.; Leng, L.; Liu, H.; Horton, J. H.; Du, L.; Gao, J.; He, C.; Qi, X.; Xu, Q.; Zhu, J. Engineering the Electronic Structure of Single‐Atom Iron Sites with Boosted Oxygen Bifunctional Activity for Zinc–Air Batteries. *Adv. Mater.* **2023**, *35*, 2209644.

(25) Li, J.; Liu, P.; Yan, J.; Huang, H.; Song, W. Fully‐Conjugated Covalent Organic Frameworks with Two Metal Sites for Oxygen Electrocatalysis and Zn–Air Battery. *Adv. Sci.* **2023**, *10*, 2206165.

(26) Wang, Y.; Zhou, T.; Ruan, S.; Feng, H.; Bi, W.; Hu, J.; Chen, T.; Liu, H.; Yuan, B.; Zhang, N.; Wang, W.; Zhang, L.; Chu, W.; Wu, C.; Xie, Y. Directional Manipulation of Electron Transfer by Energy Level Engineering for Efficient Cathodic Oxygen Reduction. *Nano Lett.* **2022**, *22*, 6622–6630.

(27) Wang, Z.; Xu, R.; Ye, Q.; Jin, X.; Lu, Z.; Yang, Z.; Wang, Y.; Yan, T.; Liu, Y.; Pan, Z.; Hwang, S.; Fan, H. J. Tailoring First Coordination Sphere of Dual‐Metal Atom Sites Boosts Oxygen Reduction and Evolution Activities. *Adv. Funct. Mater.* **2024**, *20*, 2315376.

(28) Liu, J.; Chen, W.; Yuan, S.; Liu, T.; Wang, Q. High-Coordination Fe-N4SP Single-Atom Catalysts via the Multi-Shell Synergistic Effect for the Enhanced Oxygen Reduction Reaction of Rechargeable Zn-Air Battery Cathodes. *Energy Environ. Sci.* **2023**, *17*, 249–259.

(29) Liu, M.; Wang, X.; Cao, S.; Lu, X.; Li, W.; Li, N.; Bu, X. Ferredoxin‐Inspired Design of S‐Synergized Fe‐Fe Dual‐Metal Center Catalysts for Enhanced Electrocatalytic Oxygen Reduction Reaction. *Adv. Mater.* **2024**, *20*, 2309231.

(30) Tan, X.; Zhang, J.; Cao, F.; Liu, Y.; Yang, H.; Zhou, Q.; Li, X.; Wang, R.; Li, Z.; Hu, H.; Zhao, Q.; Wu, M. Salt Effect Engineering Single Fe‐N_2_P_2_‐Cl Sites on Interlinked Porous Carbon Nanosheets for Superior Oxygen Reduction Reaction and Zn‐Air Batteries. *Adv. Sci.* **2024**, *11*, 2306599.

(31) Wang, Y.; Yang, T.; Fan, X.; Bao, Z.; Tayal, A.; Tan, H.; Shi, M.; Liang, Z.; Zhang, W.; Lin, H.; Cao, R.; Huang, Z.; Zheng, H. Anchoring Fe Species on the Highly Curved Surface of S and N Co‐Doped Carbonaceous Nanosprings for Oxygen Electrocatalysis and a Flexible Zinc‐Air Battery. *Angew. Chemie Int. Ed.* **2024**, *6*, e202313034.

(32) Wang, Z.; Lu, Z.; Ye, Q.; Yang, Z.; Xu, R.; Kong, K.; Zhang, Y.; Yan, T.; Liu, Y.; Pan, Z.; Huang, Y.; Lu, X. Construction of Fe Nanoclusters/Nanoparticles to Engineer FeN_4_ Sites on Multichannel Porous Carbon Fibers for Boosting Oxygen Reduction Reaction. *Adv. Funct. Mater.* **2024**, *20*, 2315150.

(33) Liu, M.; Zhang, J.; Su, H.; Jiang, Y.; Zhou, W.; Yang, C.; Bo, S.; Pan, J.; Liu, Q. In Situ Modulating Coordination Fields of Single-Atom Cobalt Catalyst for Enhanced Oxygen Reduction Reaction. *Nat. Commun.* **2024**, *15*, 1675.

(34) Yu, X.; Lai, S.; Xin, S.; Chen, S.; Zhang, X.; She, X.; Zhan, T.; Zhao, X.; Yang, D. Coupling of Iron Phthalocyanine at Carbon Defect Site via π-π Stacking for Enhanced Oxygen Reduction Reaction. *Appl. Catal. B Environ.* **2021**, *280*, 119437.

(35) Xu, X.; Li, X.; Lu, W.; Sun, X.; Huang, H.; Cui, X.; Li, L.; Zou, X.; Zheng, W.; Zhao, X. Collective Effect in a Multicomponent Ensemble Combining Single Atoms and Nanoparticles for Efficient and Durable Oxygen Reduction. *Angew. Chemie Int. Ed.* **2024**, *63*, e202400765.

(36) Chen, J.; Huang, B.; Cao, R.; Li, L.; Tang, X.; Wu, B.; Wu, Y.; Hu, T.; Yuan, K.; Chen, Y. Steering Local Electronic Configuration of Fe–N–C‐Based Coupling Catalysts via Ligand Engineering for Efficient Oxygen Electroreduction. *Adv. Funct. Mater.* **2023**, *33*, 2209315.

(37) Zhao, X.; Chen, J.; Bi, Z.; Chen, S.; Feng, L.; Zhou, X.; Zhang, H.; Zhou, Y.; Wågberg, T.; Hu, G. Electron Modulation and Morphology Engineering Jointly Accelerate Oxygen Reaction to Enhance Zn‐Air Battery Performance. *Adv. Sci.* **2023**, *10*, 2205889.

(38) Zhang, H.; Zhang, Z.; Zhang, Z.; Li, Y.; Hou, Y.; Liu, P.; Xu, B.; Zhang, H.; Liu, Y.; Guo, J. Highly Dispersed Ultrasmall Iron Phthalocyanine Molecule Clusters Confined by Mesopore-Rich N-Doped Hollow Carbon Nanospheres for Efficient Oxygen Reduction Reaction and Zn-Air Battery. *Chem. Eng. J.* **2023**, *469*, 143996.

(39) Li, Y.; Ding, Y.; Zhang, B.; Huang, Y.; Qi, H.; Das, P.; Zhang, L.; Wang, X.; Wu, Z.-S.; Bao, X. N,O Symmetric Double Coordination of an Unsaturated Fe Single-Atom Confined within a Graphene Framework for Extraordinarily Boosting Oxygen Reduction in Zn–air Batteries. *Energy Environ. Sci.* **2023**, *16*, 2629–2636.

(40) Zhong, J.; Liang, Z.; Liu, N.; Xiang, Y.; Yan, B.; Zhu, F.; Xie, X.; Gui, X.; Gan, L.; Yang, H. Bin; Yu, D.; Zeng, Z.; Yang, G. Engineering Symmetry-Breaking Centers and d-Orbital Modulation in Triatomic Catalysts for Zinc-Air Batteries. *ACS Nano* **2024,** *18*, 5258–5269.

(41) Meng, H.; Wu, B.; Zhang, D.; Zhu, X.; Luo, S.; You, Y.; Chen, K.; Long, J.; Zhu, J.; Liu, L.; Xi, S.; Petit, T.; Wang, D.; Zhang, X.-M.; Xu, Z. J.; Mai, L. Optimizing Electronic Synergy of Atomically Dispersed Dual-Metal Ni–N_4_ and Fe–N_4_ Sites with Adjacent Fe Nanoclusters for High-Efficiency Oxygen Electrocatalysis. *Energy Environ. Sci.* **2024,** *17*, 704–716.

(42) Liang, C.; Han, X.; Zhang, T.; Dong, B.; Li, Y.; Zhuang, Z.; Han, A.; Liu, J. Cu Nanoclusters Accelerate the Rate–Determining Step of Oxygen Reduction on Fe–N–C in All pH Range. *Advanced Energy Materials*. **2024,** 14, 2303935.

(43) Lyu, L.; Hu, X.; Lee, S.; Fan, W.; Kim, G.; Zhang, J.; Zhou, Z.; Kang, Y. M. Oxygen Reduction Kinetics of Fe–N–C Single Atom Catalysts Boosted by Pyridinic N Vacancy for Temperature-Adaptive Zn–Air Batteries. *J. Am.Chem. Soc.* **2024,** *146*, 4803-4813.
